# Disruption of pH Dynamics Suppresses Proliferation and Potentiates Doxorubicin Cytotoxicity in Breast Cancer Cells

**DOI:** 10.3390/pharmaceutics13020242

**Published:** 2021-02-09

**Authors:** Diana Tavares-Valente, Bárbara Sousa, Fernando Schmitt, Fátima Baltazar, Odília Queirós

**Affiliations:** 1 Life and Health Sciences Research Institute (ICVS), School of Medicine, University of Minho, 4710-057 Braga, Portugal; dvalente@porto.ucp.pt; 2 PT Government Associate Laboratory, ICVS/3B’s—Life and Health Sciences Research Institute/Biomaterials, Biodegradables and Biomimetics, 4710-057 Braga/Guimarães, Portugal; 3 Department of Sciences, IINFACTS—Institute of Research and Advanced Training in Health Sciences and Technologies, University Institute of Health Sciences (IUCS), CESPU, CRL, 4585-116 Gandra, Portugal; 4 IPATIMUP—Institute of Molecular Pathology and Immunology of the University of Porto, University of Porto, 4200-135 Porto, Portugal; bsousa@ipatimup.pt (B.S.); fschmitt@ipatimup.pt (F.S.); 5 i3S—Institute for Research & Innovation in Health, University of Porto, 4200-135 Porto, Portugal; 6 FMUP—Faculty of Medicine of the University of Porto, University of Porto, 4200-319 Porto, Portugal

**Keywords:** pH regulators, reverse pH gradient, tumor microenvironment, treatment resistance

## Abstract

The reverse pH gradient is a major feature associated with cancer cell reprogrammed metabolism. This phenotype is supported by increased activity of pH regulators like ATPases, carbonic anhydrases (CAs), monocarboxylate transporters (MCTs) and sodium–proton exchangers (NHEs) that induce an acidic tumor microenvironment, responsible for the cancer acid-resistant phenotype. In this work, we analyzed the expression of these pH regulators and explored their inhibition in breast cancer cells as a strategy to enhance the sensitivity to chemotherapy. Expression of the different pH regulators was evaluated by immunofluorescence and Western blot in two breast cancer cell lines (MDA-MB-231 and MCF-7) and by immunohistochemistry in human breast cancer tissues. Cell viability, migration and invasion were evaluated upon exposure to the pH regulator inhibitors (PRIs) concanamycin-A, cariporide, acetazolamide and cyano-4-hydroxycinnamate. Additionally, PRIs were combined with doxorubicin to analyze the effect of cell pH dynamic disruption on doxorubicin sensitivity. Both cancer cell lines expressed all pH regulators, except for MCT1 and CAXII, only expressed in MCF-7 cells. There was higher plasma membrane expression of the pH regulators in human breast cancer tissues than in normal breast epithelium. Additionally, pH regulator expression was significantly associated with different molecular subtypes of breast cancer. pH regulator inhibition decreased cancer cell aggressiveness, with a higher effect in MDA-MB-231. A synergistic inhibitory effect was observed when PRIs were combined with doxorubicin in the breast cancer cell line viability. Our results support proton dynamic disruption as a breast cancer antitumor strategy and the use of PRIs to boost the activity of conventional therapy.

## 1. Introduction

Breast cancer is the most common cancer in the female gender and, according to Globocan, about 2.1 million new cases of breast cancer were identified in 2018 in the world and over 627,000 women died from the disease (global health estimates, World Human Organization (WHO)) [1]. Breast cancer is characterized by distinct biological features and subdivided into different molecular subtypes [2,3]. One of the most aggressive subtypes is the basal-like, which belongs to the triple-negative breast cancer (TNBC) type, being negative for estrogen receptor (ER) (-), human epidermal growth factor receptor 2 (HER2) and progesterone receptor (PgR). TNBC has a small therapeutic window, with low rates of treatment success [4,5]. Unfortunately, few treatment options are available besides chemotherapy. The common regimens include anthracyclines, cyclophosphamide, and taxanes, with possible serious side-effects [6]. Therefore, new and more effective therapies are urgently needed. Different studies with novel therapies are ongoing such as hormonal and immunotherapy. Genetic therapy is also being studied as a new treatment modality for this type of breast cancer. Al-Mahmood et al. proposed that the codelivery of liposomes loaded with EGFR targeted siRNA and gefitinib, respectively, suppresses resistance of TNBCs to gefitinib, increasing the effectiveness of the treatment [7].

Highly proliferative cancer cells reprogram their metabolism, being mainly dependent on glycolytic metabolism instead of oxidative phosphorylation (OXPHOS) to produce ATP, even in aerobic conditions, being this finding called ‘‘aerobic glycolysis” or “Warburg effect” [8]. The hyper-glycolytic phenotype of cancer cells has, as a consequence, chronic acidification of the microenvironment due to the increase in proton efflux. Therefore, the tumor microenvironment presents an acidic pH between 6.5 and 7.1, in contrast to normal tissues, where the extracellular pH (pHe) is between 7.2 and 7.5 [9]. To avoid cell death due to intracellular acidification, cancer cells upregulate different families of pH regulators at the plasma membrane (PM) [10,11]. These proteins include the carbonic anhydrase (CA) family, specifically CAIX and CAXII, the sodium-hydrogen exchangers (NHEs), namely NHE1, the vacuolar-ATPase (V-ATPase) and monocarboxylate transporters (MCTs), MCT1 and MCT4 in particular [10,12]. This phenotype provides a competitive advantage to cancer cells since the low pHe generates a microenvironment favorable to tumor invasion and metastases, angiogenesis, suppression of anticancer immune response and resistance to chemo- and radiotherapy [10,13,14,15]. Some reports demonstrated the association between pH regulator expression and some breast cancer subtypes, such as V-ATPase and CAIX expression in breast basal-like cancer [16,17], and V-ATPase and NHE1 expression in HER2 positive and luminal breast cancers [18,19]. Further, expression of GLUT1, MCT1 and CD147 has been connected with high-grade tumors [20] and the expression of MCT4 with TNBC [21].

The interaction between tumor cells and the surrounding microenvironment motivated the exploitation of pH regulators as targets to improve anticancer therapy. Many compounds have been developed to inhibit the activity of the pH regulators mentioned above [12]. Non-amiloride compounds, such as cariporide, are specific and powerful NHE1 inhibitors [22,23]. The use of cariporide was reported to induce a reduction in cholangiocarcinoma [24] and human tongue squamous cell carcinoma [25] cell proliferation. Although it was well tolerated by humans in the context of cardiac disease, there are still no studies in the cancer field. Regarding CAs, namely CAXII, accumulating evidence recognizes that CA inhibition successfully decreases tumor growth in in vitro and in vivo models [26,27,28,29]. Supuran and coworkers demonstrated that the sulfonamide family of compounds impairs tumor growth and invasion in breast cancer models, being already in the clinical trial phase. A phase I clinical trial with LC-0111(NCT02215850), a small molecule inhibitor of CAIX, showed the compound as being safe and appropriate to start phase 2 [30].

Bafilomycin and concanamycin A (conc. A), which belong to the plecomacrolide class of antibiotics, decreased the invasion capacity of breast cancer cells [31] by specific inhibition of V-ATPase. However, there are only a few reports on the effect of these compounds on cancer cells. Concerning MCT inhibition, Morais-Santos et al. demonstrated that MCT inhibition, by siRNA or pharmacologic inhibition with cyano-4-hydroxycinnamate (CHC), lodinamine or quercetin, decreased breast cancer cell aggressiveness in vitro [32]. Furthermore, the MCT knockdown decreased in vivo tumor formation and growth [33]. The importance of MCT inhibition in cancer therapy also has been revealed in different types of cancer, both in in vitro and in vivo models [34], namely in colorectal [35] and cervix [36] cancers and glioma [37,38].

The acidic tumor microenvironment confers many advantages to cancer cells; therefore, blockade of proton export via the inhibition of pH regulators is a promising strategy in cancer therapy. Some pH regulator inhibitors (PRIs) were by now tested in different phases of clinical trials [39]. However, further efforts are needed to increase the number of studies aiming to clarify the efficacy and possible toxicity generated by some of these compounds in specific cancer models. Therefore, this study aimed to disrupt the pH regulatory armamentarium of breast cancer cells and understand how this can be used to improve current therapy.

## 2. Material and Methods

### 2.1. Breast Tumor Samples

Tissue microarrays (TMAs) were constructed with samples from 473 patients with invasive breast carcinomas retrieved from the histopathology files of three Departments of Pathology: University Hospital of the Federal University of Santa Catarina (UFSC, Florianópolis, Brazil), Hospital Divino Espírito Santo (Ponta Delgada, Portugal) and from a private Laboratory of Pathology (Veronese Patologia e Citologia Araçatuba, Brazil), being the samples evaluated for clinical and pathological features (Additional file Table S1). Tissue molecular characterization was performed as previously described [40]. This study was approved by the UFSC Ethics Committee for Human Research (CEPSH), by the Ethics Committee for Health from the Hospital do Divino Espírito Santo de Ponta Delgada E.P.E., and by the research review boards from the Veronese Patologia e Citologia Araçatuba Pathology Laboratory, according to the national regulative law and ethical guidelines, concerning the handling of biological specimens from tumor banks. The samples were exclusively accessible for research purposes in retrospective studies, in accordance with the international declaration of Helsinki.

### 2.2. Cell lines and Culture Conditions

The breast cancer cell lines MCF-7 (ER +) and MDA-MB-231 (ER −) [41] used in this work were obtained from American Type Culture Collection (USA) and were tested for mycoplasma contamination (quantitative PCR) just before the assays. Cells were cultured according to ATCC recommendations, in Roswell Park Memorial Institute medium 1640 (RPMI 1640), supplemented with 10% heat-inactivated fetal bovine serum (FBS), 1% penicillin–streptomycin solution, in a 37 °C humidified atmosphere with 5% CO_2_.

### 2.3. Drugs

Stock solutions of 40 mM doxorubicin (Sigma-Aldrich; Algés, Portugal) and of the PRIs, 30 mM concanamycin. A (Conc. A) (Biotechnology, Santa Cruz, Dallas, TX, USA), 1 mM α-cyano-4-hydroxycinnamate (CHC), 0.5 cariporide and 100 mM acetazolamide (AZ) (Sigma-Aldrich, Algés, Portugal) were dissolved in dimethyl sulfoxide (DMSO). The working solutions were prepared by dilution in a cell culture medium (complete RPMI). Controls with DMSO without drugs were used. All the solutions were filtered through a 0.2 µm filter before use.

### 2.4. Cell Survival Assays

Cell viability was assessed by the sulforhodamine B (SRB) assay (Sigma-Aldrich, Algés, Portugal). SRB is a bright purple dye with two sulfonic groups, soluble in water, which binds to the basic amino acid residues of cell proteins. The dye SRB can then be used as a quantitative indicator of cell protein content, which is proportional to the cell density. An alteration in cell number originates from a proportional change in the amount of dye incorporated, being associated with the degree of cytotoxicity of the compound under study [42].

Cells were plated into 96-well plates, at a density of 1.5 × 10^4^ cells/well, and treated 48 h with the chemotherapeutic drug doxorubicin and the different PRIs. In each assay, the compounds were tested in triplicate. Untreated cells were used as a control, with the vehicle employed to prepare the drug (DMSO) used at the same concentration of the assays. Wells without cells, but filled with the same volume of culture medium containing the different concentrations of compounds, were used as negative controls and tested in duplicate. After cell incubation at appropriate conditions in 96-well plates (100 μL/well), cells were fixed with 50% cold trichloroacetic acid (25 μL/well) added directly to the culture, for 1 h at 4 °C. TCA was then removed by rinsing the plates 5 times with distilled water. Cells were then air-dried and stained with 0.4% SRB solution (50 μL/well), during 30–60 min at 37 °C, and the plates were again rinsed 5 times with 1% acetic acid until the unincorporated dye was totally removed. The plates were dried overnight, and the incorporated SRB was solubilized in a 10 mM Tris base solution (100 μL/well). The absorbance was measured at OD540 nm, using a microplate reader.

IC_50_ values were estimated from the GraphPad software, using nonlinear regression of sigmoidal dose–response after logarithmic transformation.

### 2.5. Western Blotting

Breast cancer cell lines were incubated until reaching 80% confluence, homogenized and centrifuged. The supernatants were then collected, and total protein quantified (Pierce™ BCA protein assay kit). 25 µg of total protein from each cell line were separated by SDS–PAGE on 10% polyacrylamide gels and transferred onto nitrocellulose membranes using a 25 mM Tris-base/glycine buffer. Membranes were blocked with 5% milk in TBS/0.1% Tween for 1 h at room temperature and probed overnight at room temperature with primary antibodies at the appropriate dilution (see Table 1). After the incubation period, the membranes were washed and incubated with the adequate secondary antibodies coupled to horseradish peroxidase (anti-rabbit, A9169, Sigma; anti-mouse, PI-2000, Vector) for 1 h at room temperature. Protein quantification through band densitometry determination was performed after image acquiring, using ImageJ software (version 1.41, National Institutes of Health). α-tubulin was used as a loading control.

### 2.6. Immunofluorescence

MDA-MB-231 and MCF-7 cells (3 × 10^5^ cells/well) were plated on glass coverslips, fixed with cold methanol, permeabilized with triton 0.1% diluted in PBS 1× and blocked with BSA (bovine serum albumin) 5%, 30 min at room temperature. Cells were then first incubated with the respective primary antibodies (Table 1) at room temperature overnight and then with the secondary antibody anti-rabbit-Alexa Fluor 488 (1:1000 dilution, A11008, Invitrogen™ Molecular Probes™) and anti-mouse-Alexa Fluor 488 (1:1000 dilution, A11001, Invitrogen™ Molecular Probes™), diluted in BSA 5%, for 1 h at room temperature. Finally, cells were mounted in Vectashield mounting media with 4′,6-diamidino-2-phenylindole (DAPI) and the photographs were captured with a fluorescence microscope (Zeiss Spinning Disc AxioObserver Z.1 SD microscope coupled to an AXIOCam MR3 Camera) and treated with ImageJ software (National Institute of Health, Bethesda, MD, USA).

### 2.7. Immunohistochemistry

Immunohistochemistry was performed as previously described [43]. The primary antibodies used were as follows: rabbit anti-V-ATPase (1:100, ab157458 Abcam) and rabbit anti-CAXII (1:200, ab195233, Abcam). IHC was performed with the Ultravision detection system anti-polyvalent, HRP (Lab Vision Corporation). After deparaffinization and rehydration, the slides were heated for 20 min at 98 °C with 10 mM citrate buffer (pH 6.0) for antigen retrieval. Endogenous peroxidase was inactivated, and the tissues incubated with the primary antibody for 2 h at room temperature. The immune reaction was visualized using the chromogen 3,3′-diaminobenzidine (DAB+ substrate system; Dako). Tissue sections were counterstained with Gill-2 hematoxylin. For negative controls, primary antibodies were replaced by a universal negative control antibody (N1699, Dako). Positive controls were normal colon tissue for CAXII and breast cancer tissue for V-ATPase. Immunoreaction was evaluated using a semiquantitative score system, considering the extension and intensity of staining, as previously described [43]. Some of the cases could not be evaluated for immunoreaction, due to missing TMA samples or to insufficient representation of the tumor.

### 2.8. Wound-Healing Assay

Cell migration was evaluated through the wound-healing assay. Breast cancer cells were inoculated in 6-well plates at a density of 1.0 × 10^6^ cells/well until reaching full confluence. In the confluent cells, the wounds were created manually with a yellow tip. Cells were gently washed with PBS and exposed to media without FBS with different drug concentrations. Untreated cells were used as control. The absence of FBS assures that cell migration is observed and not cell proliferation. At three different time points (0, 12 and 24 h) and for each wound, two sites were photographed at 100× magnification in an inverted microscope (Nikon Eclipse TE 2000-U, Amsterdam, Netherlands). The migration distances were determined with the MeVisLab platform, and the percentage of cell migration was calculated with the GraphPad Prism 4 software (USA).

### 2.9. Invasion Assay

Cell invasion was performed using 24-well BD Biocoat Matrigel invasion chambers, according to the manufacturer’s instructions (354,480, BD Biosciences) [44]. Matrigel invasion chamber was rehydrated, and the cells were there plated and incubated with the IC_50_ concentrations of the different PRI for a period of 24 h. The invading cells were fixed with methanol and stained with Gill hematoxylin. The number of invading cells was determined using the Image J software (version 1.41; National Institute of Health, USA) in the membranes photographed with an Olympus SZx16 stereomicroscope (16×). Invasion was calculated as the percentage of cell invasion normalized for the control condition.

### 2.10. Effect of the pH Regulator Inhibitors on Doxorubicin Cytotoxicity

Breast cancer cells were plated at a density of 1.5 × 10^3^ cells/well into 96-well plates. Cells were treated with the IC_50_ of each PRI (this value was chosen because it has been determined in previous assays that were the concentration that induced some positive effect when combined with doxorubicin) combined with different concentrations of doxorubicin (range between 0.001 and 100 µM) for 48 h. The effect of doxorubicin alone and PRI + doxorubicin on cell growth was evaluated by the SRB assay. The combined effect of the drugs was determined using the CalcuSyn software (Biosoft), which is based on the Chou–Talalay median-effect equation derived from the mass-action law principle. According to this principle, increasing drug concentration will increase its potency, giving a measurable dose–response and effect on cellular synergy, being suitable to assay drug–drug interaction. Each drug combination (doxorubicin and each PRI) was evaluated at different drug ratios (nonconstant ratios with a fixed PRI concentration (IC_50_) and variable doxorubicin concentrations), as described in the results, to identify the presence of synergy. Cell viability data were introduced into the program, as well as the concentration ratios, in order to determine the combination index (CI). The resulting CI determined by the software allows the quantitative determination of drug interactions, namely additive effect (CI = 1), synergism (CI < 1), and antagonism (CI > 1) in drug combinations [45].

### 2.11. Zymography Assay

To analyze metalloproteinase 2 and 9 (MMP-2 and MMP-9) activities, a zymography assay was performed using a 10% polyacrylamide gel containing 0.1% of gelatine as substrate. The media from the breast cancer cell lines exposed to different extracellular pH s and treated with PRI were collected and centrifuged before use. Briefly, 20 µg of protein was loaded in the stacking gel, and after electrophoresis, gels were washed with 2% Triton X-100 solution to remove the SDS remnants, incubated for 16 h at 37 °C in appropriate buffer with MMP substrate and stained with Coomassie brilliant blue solution for 30 min. The excess stain was removed by a solution of methanol and acetic acid. Gels were photographed, and the protease activity was estimated using ImageJ software (version 1.41, National Institutes of Health).

### 2.12. Statistical Analysis

The GraphPad Prism 5 software was used for statistical analysis, and the results presented as normalized means ± SD for n independent experiments. Significant differences between groups: * *p* < 0.05; ** *p* < 0.01; *** *p* < 0.001 compared to untreated cells (control). Ns: non-significant.

Concerning the immunohistochemistry results, statistical analysis was carried out using the SPSS statistics 17.0 software (SPSS Inc., New York, NY, USA). Pearson’s chi-squared (χ2) test and contingency tables were used to determine associations between groups.

## 3. Results

### 3.1. pH Regulators Are Strongly Expressed in Breast Cancer Cell Lines, but with a Distinct Expression Pattern

**Figure 1:**
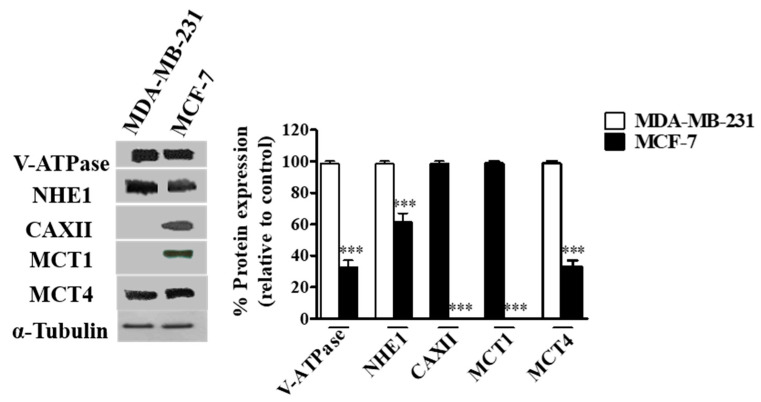
Protein levels in breast cancer cells assessed by Western-Blot. A representative blot is shown. Results are expressed as mean ± SD of triplicates from three independent experiments. The cells with the highest expression of each pH regulator were used as reference and normalized to 100. *** *p* < 0.001 compared to reference cells.

The expression of the different pH regulators (MCT1, MCT4, CAXII, V-ATPase and NHE1) was assessed in the breast cancer cell lines MCF-7 and MDA-MB-231 by Western blot. Expression was positive for all pH regulators in both cell lines, except for MCT1 and CAXII in MDA-MB-231 cells (MCT1 is silenced in these cells due to promoter methylation [46]) (Figure 1). The expression of V-ATPase (subunit A1), MCT4 and NHE1 was higher in MDA-MB-231 cells (Figure 1). Concerning cell localization, V-ATPase and NHE1 were found to be expressed at the plasma membrane (PM), whereas their expression in MCF-7 cells was mainly localized in the cytoplasm. Regarding lactate transporters, both MCT1 and MCT4 were expressed both at the PM and cytoplasm (MCT4 in both cell lines, MCT1 only in MCF-7 cells). Expression of CAXII was only detected in MCF-7 cells, and it was mainly present in cell contact-junctions (Additional file Figure S1).

### 3.2. V-ATPase Overexpression Associates Significantly with High-Grade Invasive Breast Carcinomas

In this study, we used 473 invasive breast carcinomas, and 48 normal breast tissues in TMAs previously classified for molecular subtypes [38] to characterize the expression of the pH regulators CAXII and V-ATPase.

**Figure 2:**
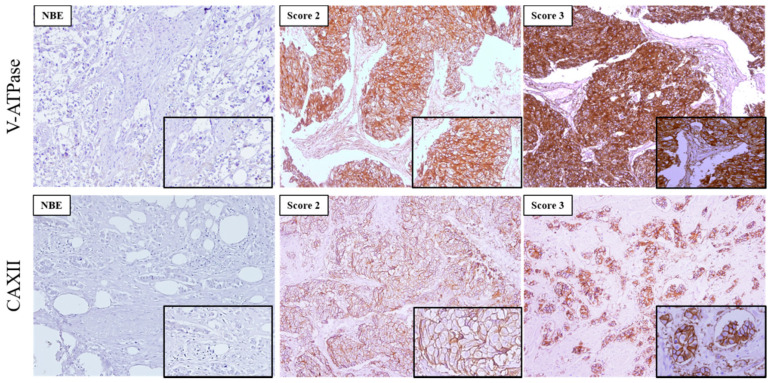
Immunoexpression of V-ATPase and CAXII in primary invasive breast carcinomas and normal breast epithelium (NBE). Images are in 100× magnification, and the insets are in 200× magnification. The score for immunoreactive extension was score 2:5–50% and score 3: >50% of immunoreactive cells.

The expression of the two pH regulators was observed both at the PM and cytoplasm. V-ATPase was more frequently found in the cytoplasm, with different immunoreactivity extension (Figure 2, Table 2). Most cases expressed V-ATPase in the cytoplasm (197/203, 97%), with 51/197 (25.9%) of cases positive for PM expression (Table 2). Regarding CAXII, 50% (98/196) of the cases were positive, and membrane expression was found in 98.0% (96/98) of the positive cases. PM expression of CAXII and V-ATPase was significantly higher in breast cancer samples in comparison to normal breast tissue (*p* = 0.001; *p* = 0.046, respectively) (Table 2). Specifically, normal breast tissue did not express CAXII, and only 4/44 (9.1%) cases were positive for V-ATPase at the PM. Representative IHC pictures for both pH regulators can be observed in Figure 2.

In order to study the relationship between V-ATPase and CAXII and MCT1 or MCT4 expressions in the tumor samples, we looked for association among the expression of these proteins (Table 3). Due to the role of these proteins, only plasma membrane expression was considered. Overall, no significant associations were found between V-ATPase and MCT1 or MCT4 expressions. Moreover, we did not find associations between CAXII and MCT1 or MCT4.

The association between the expression of each of these proteins with breast cancer molecular characteristics and molecular subtypes (Additional file Table S2), as well as with available specific metabolic biomarkers (HIF1, GLUT1, CAIX and CD147) (Additional file Table S3), was evaluated. V-ATPase expression was differentially expressed in the molecular subtypes of breast cancer (*p* = 0.006), with higher expression in the luminal A subtype (Additional file Table S2). Additionally, we observed a significant positive association between V-ATPase and ER, PgR and HER2 (*p* = 0.001, *p* = 0.006 and *p* = 0.003, respectively) (Additional file Table S2). However, no significant association was observed between V-ATPase expression and classical breast cancer prognostic factors, namely lymph node (LN) invasion. In addition, no significant association with the metabolic biomarkers was found (Additional file Table S3). Regarding CAXII, we also observed a different expression pattern among the molecular subtypes (*p* = 0.010), but in this case, the highest expression was in the basal subtype (Additional file Table S2). It is important to note that 38.0% of cases positive for CAXII presented LN invasion (*p* = 0.010). Further, we found a correlation between CAXII and PgR expression (*p* = 0.010) (Additional file Table S2). Similar to V-ATPase, no association was found with the metabolic biomarkers. However, CAXII expression was associated with V-ATPase PM expression (*p* = 0.030) (Additional file Table S3).

### 3.3. Extracellular Acidity Decreases Sensitivity to Doxorubicin and Increases the Migratory and Invasive Abilities of Breast Cancer Cells

**Figure 3:**
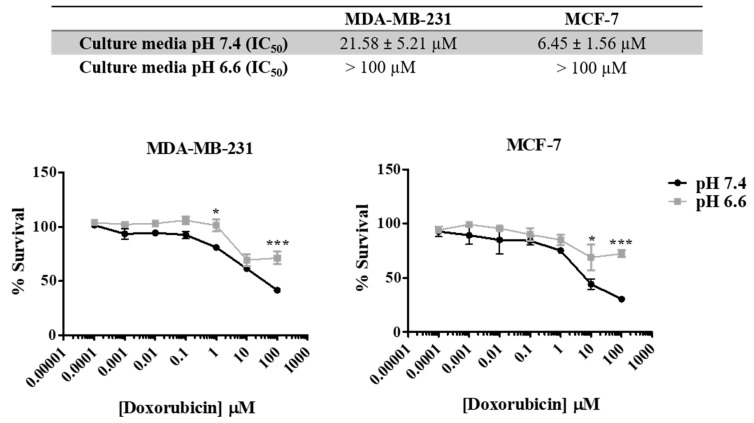
Effect of doxorubicin on breast cancer cell survival at different extracellular pH values. IC_50_ values were determined after drug exposure using the SRB assay. Results are expressed as mean ± SD of triplicates from at least three independent experiments. Medium at pH 7.4 was used as control. * *p* < 0.05; *** *p* < 0.001 compared to pH 7.4 (control).

As previously described, the acidic pH in the extracellular space is responsible for aggressive tumor features, including drug resistance. In Figure 3, we show that both cell lines present different sensitivities to doxorubicin in media with different pH s (7.4 vs. 6.6). pH was controlled by buffering RPMI medium with 25 mM HEPES. The increase in the extracellular acidity (pH 6.6) decreased cancer cell sensitivity to doxorubicin (48 h), as can be seen by the lower IC_50_ values for the physiologic pHe of 7.4 (21.58 µM for MDA-MB-231 and 6.45 µM for MCF-7) compared to the acidic pHe of 6.6 (>100 µM for both cell lines).

**Figure 4:**
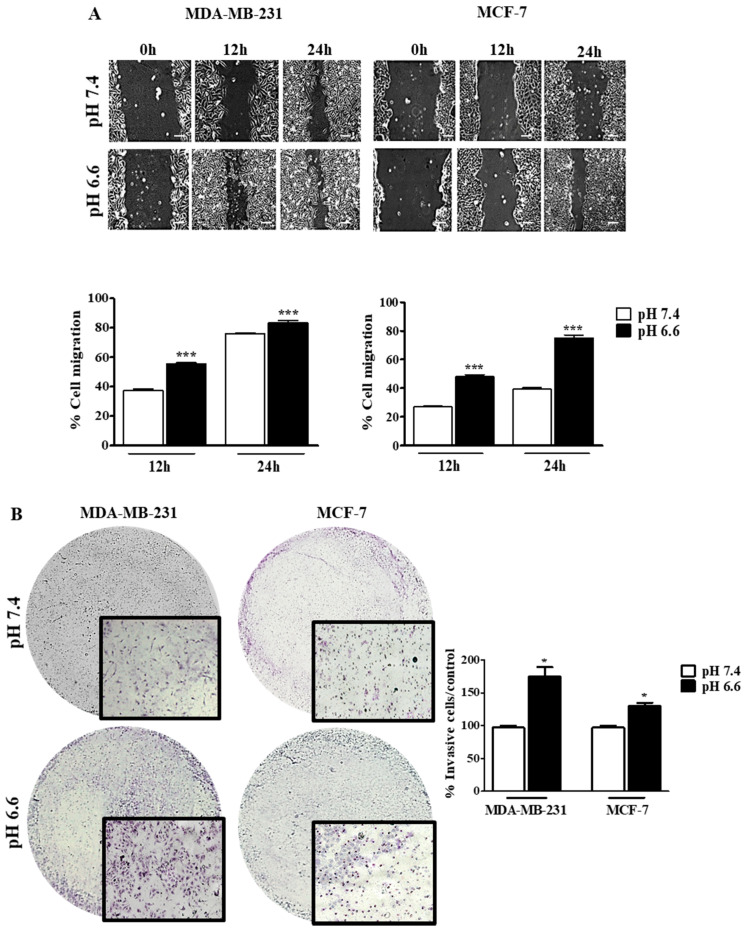
Cell migration and invasion of breast cancer cell lines at different extracellular pH values. Cell migration (**A**) and cell invasion (**B**) were evaluated by the wound-healing and Matrigel assays, respectively. In the wound-healing assay, the scale bar corresponds to 100 μm. Results are expressed as mean ± SD of triplicates from three independent experiments. Medium at pH 7.4 was used as control. * *p* < 0.05; *** *p* < 0.001 compared to pH 7.4 control.

The effect of pHe on cell migration was studied by the wound-healing assay at both pH values, and the migration of tumor cells was registered at different time points (0, 12 and 24 h) (Figure 4). There was an increase in the migratory ability in both cell lines at pH 6.6, in comparison to pH 7.4. However, this difference in migration was more evident in MCF-7 than MDA-MB-231, probably because the latter cells already present a high ability to migrate in basal conditions (Figure 4A). Moreover, we evaluated the invasion ability of these cells in a low extracellular pH environment. The decrease of pHe also led to an increase of cell invasion in both cell lines, as can be seen by the number of cells that migrate through the Matrigel membrane. This difference was higher for MDA-MB-231 cells compared to MCF-7 cells (Figure 4B). Further, this effect is probably mediated by MMP9 and MMP2 since we observed higher activity of these MMPs at lower pHe in both cell lines. Accordingly, this effect was more evident in MDA-MB-231 (Additional file Figure S2).

### 3.4. Disruption of pH Regulation Decreases the Aggressive Behavior of MDA-MB-231 Cells

**Figure 5:**
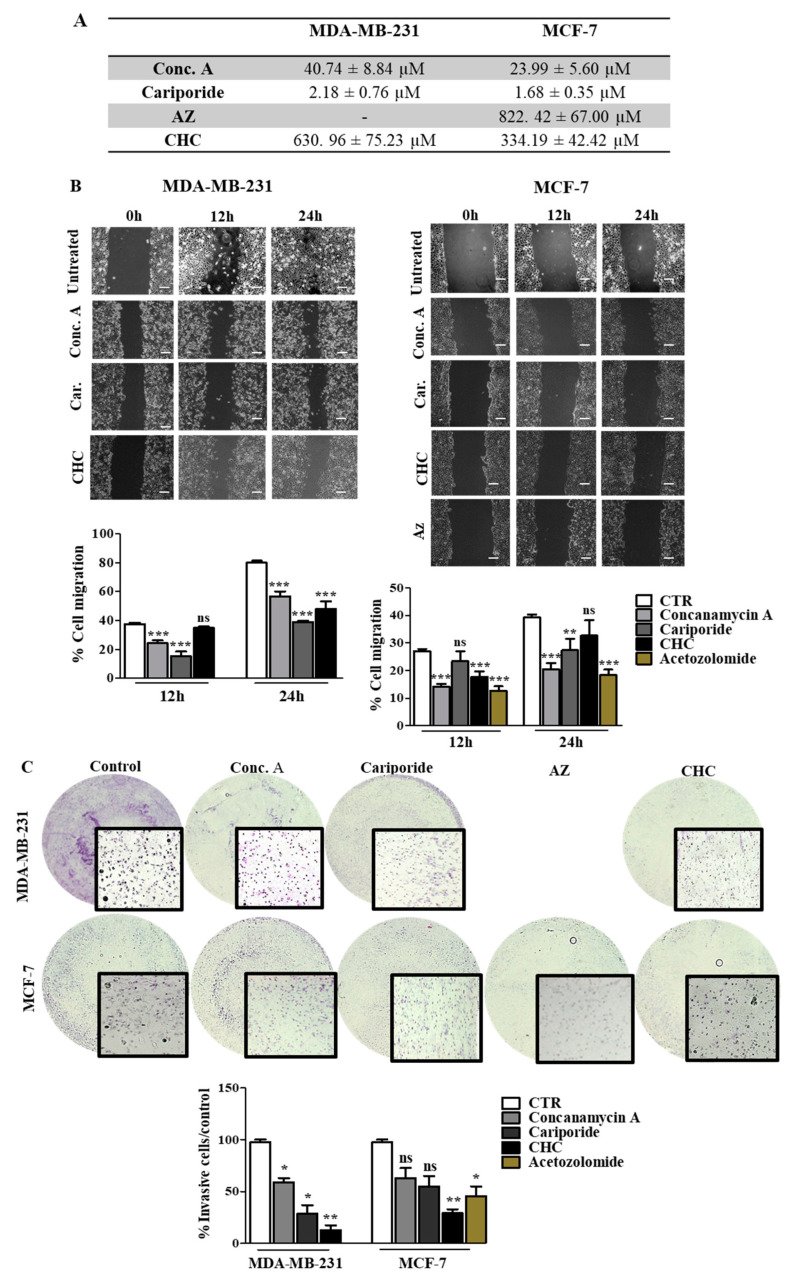
Effect of pH regulator inhibitors (PRI) on breast cancer cell features. (**A**) IC_50_ (concentration of drug required for 50% of growth inhibition) values determined after PRI exposure, using the sulforhodamine B (SRB) assay. (**B**,**C**) Cell migration and invasion analysis were done after 24 h of treatment with IC_50_ values of PRI determined in (**A**). Representative images of the migration assay at 0, 12 and 24 h and of the invasion assay at 24 h, are presented. In the wound-healing assay, the scale bar (white bar) corresponds to 100 μm. Results are expressed as the mean + SD of at least 3 independent experiments, each in triplicate. Untreated cells were used as control. * *p* < 0.05; ** *p* < 0.01; *** *p* < 0.001 compared to untreated cells; ns—non-significant.

Our results support the relevance of pH regulation in the aggressive features of cancer cells, and therefore the inhibition of the main players in this phenotype can be a useful strategy to decrease tumor aggressiveness. Thus, we inhibited the different pH regulators with specific compounds (pH regulators inhibitors—PRIs) in both cell lines. First, all PRIs were able to decrease cell survival in both cell lines. Conc. A and cariporide were the compounds that induced the highest cytotoxic effect, with lower IC_50_ values for both cell lines (Figure 5A). The effect of pH regulation disruption on cell migration and invasion was more evident in MDA-MB-231 cells (Figure 5B,C). At 24 h, CHC, cariporide and conc. A were able to decrease cell migration and invasion significantly through the Matrigel matrix in these cells. In contrast, MCF-7 cells demonstrated a lower migratory and invasive capacity after 24 h. Nevertheless, all the PRIs, except for CHC, decreased cell migration (Figure 5B), but only CHC and AZ were able to decrease MCF-7 cell invasion (Figure 5C). AZ compound was just assayed in MCF-7 cells, as CAXII was expressed only in this cell line. Further, the activity of MMPs, namely MMP-2, demonstrated by the gel digestion in the zymography assay, was lower in the presence of the PRIs, in MDA-MB-231 cells (Additional file Figure S3). In MCF-7 cells, all PRIs also inhibited MMP-2 activity in a significant way, except for cariporide. Regarding MMP-9, the zymography gel showed low activity in both cell lines. Nevertheless, the% of MMP digestion was significantly lower in both cells in the presence of the PRIs.

### 3.5. Treatment with pH Regulator Inhibitors Increased the Sensitivity of Breast Cancer Cell Lines to Doxorubicin

**Figure 6:**
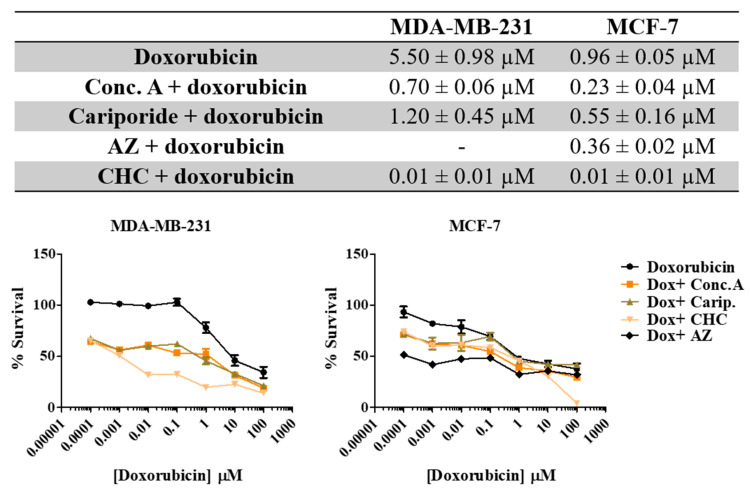
Effect the combination of PRI with doxorubicin in breast cancer cell survival. IC_50_ values were determined after 48 h of exposure with the combined treatment with a range of doxorubicin concentrations (0.0001–100 µM) and the respective PRI IC_50_, using the SRB assay. Representative graphs show the dose–response curves of the combined treatment and doxorubicin treatment alone. Results are expressed as mean ± SD of triplicates from at least three independent experiments.

Here we aimed to evaluate the value of the combination of the PRIs with the conventional drug doxorubicin in breast cancer cells. Thus, we exposed both cell lines to different combinations of both drugs for 48 h and evaluated cell viability by the SRB assay (Figure 6). All the PRI compounds enhanced doxorubicin cytotoxicity, inducing a synergistic decrease in cell survival in both cell lines. This effect was more evident in MDA-MB-231 cells, as the initial IC_50_ value determined for doxorubicin alone was higher (IC_50_ = 5.5 µM) than MCF-7 cells (IC_50_ = 0.96 µM). The MCT inhibitor CHC was the compound that induced the highest effect in both cell lines when combined with doxorubicin. Simultaneous treatment with acetazolamide (AZ) was able to reduce cell MCF-7 viability. The CalcuSyn software (Biosoft) was used to analyze the power of these combinations (Table 4). Conc. A and cariporide led to a synergistic effect (CI < 1) with doxorubicin in both cell lines, but only when using IC_50_ or IC_75_ of both drugs. CHC and AZ presented a combination index lower than 1 for all drug ICs, indicating a synergistic effect. In summary, both cell lines became considerably more sensitive to doxorubicin when exposed to the combined treatment with PRIs.

## 4. Discussion

Conventional antitumor treatments often present a low-term efficacy due to the development of multidrug resistance (MDR), the mechanism by which cancers develop resistance to multiple chemotherapeutic drugs [47]. Thus, it is important to explore new therapeutic strategies to enhance the efficacy of the available therapies. Although the influence of hypoxia and angiogenesis on tumor characteristics has been greatly explored in the last years, the role of other microenvironmental stresses is still an open field, namely the altered metabolism and the consequent acidity of the extracellular milieu.

Cancer cells commonly exhibit the “Warburg effect”, with increased glycolysis rates and lactic acid production. To prevent apoptosis by intracellular acidification, cancer cells upregulate different pH regulators at the PM, creating an acidic tumor microenvironment, also associated with chemoresistance [13]. Therefore, pH regulators, which play a key role in the reversion of the pH gradient, are interesting targets to potentiate the therapeutic efficacy of anticancer agents [48]. The main objective of this work was then to investigate the relationship between tumor extracellular acidification and the efficacy of conventional drugs in cancer cells by exploring the role of known pH regulators. Upregulation of glycolysis results in an increased production of lactic acid, leading to chronic acidification of the local environment (pH 6.5–7.1), which is fatal to the surrounding normal cells. To avoid apoptosis, cancer cells develop a self-defense strategy by upregulating the membrane pH regulators, keeping an intracellular pH (pHi) ranging from neutral to slightly alkaline (>7.2) [10].

The multiple families of pH regulators present in the PM of cancer cells are co-expressed and redundant. These include CAIX and CAXII (which hydrate extracellular CO_2_, an additional source of acidity), the anion exchanger (AE, exchanges Cl^−^ by HCO_3_^−^, contributing to intracellular buffering) and the proton exporters NHE1, Vacuolar-ATPase (V-ATPase) and MCTs, in particular, MCT1 and MCT4 [49,50]. However, these transporters are not simultaneously upregulated in cancer cells. This is a defense mechanism developed by cancer cells to compensate for the inhibition of one transporter, in which they upregulate other(s) in order to maintain the abnormal cellular alkalinity [51].

First, we evaluated the expression of pH regulators in different breast cancer cell lines and associated this expression with the sensitivity of the cells to antitumor drugs. Different reports showed the importance of pH regulation in treatment resistance due to ion-trapping. Most chemotherapeutic drugs, including anthracyclines, anthraquinones and vinca alkaloids commonly used in chemotherapy regimens (mitoxantrone, doxorubicin, daunorubicin and vinblastine), are weak lipophilic bases with pKa values between 7.4 and 8.4. The ion-trapping model predicts that this kind of drug will concentrate in more acidic compartments, namely extracellular space and some cytoplasmic organelles like lysosomes, endosomes, the trans-Golgi network and secretory vesicles [47,52]. In acidic conditions, these drugs became charged (ionized form), being their transport across the PM inhibited, and their cytoplasmic accumulation decreased, leading to lower cytotoxicity [53]. Doxorubicin is widely used, alone or in combination with other drugs, in the treatment of several cancer types, including breast cancer [54]. MDA-MB-231 cells present a lower sensitivity to doxorubicin than MCF-7 cells (higher IC_50_ value). These results are in agreement with the literature, where it is described that doxorubicin is more effective in inducing apoptosis in MCF-7 than in MDA-MB-231 cells [55]. Both breast cancer cell lines presented positive expression of the pH regulators studied, namely V-ATPase, NHE1 and MCT4 at both cytoplasm and PM. However, only MCF-7 cells presented expression of both MCT1 and CAXII.

The involvement of these proteins, as well as the acidic tumor environment in the acquisition of resistance to therapies, has been extensively described [10,12]. Accordingly, our results showed that at lower pHe (medium buffered to pH 6.6), both cell lines presented increased IC_50_ values, more evident for MCF-7 that became less sensitive to treatment. Many common drugs used in chemotherapy are weak bases, namely anthracyclines like doxorubicin, which become protonated at low pHe, as the one often present in the tumor microenvironment or in intracellular vesicles [56]. Besides the decrease in sensitivity to doxorubicin at low pHe, it is important to highlight that the IC_50_ values for doxorubicin in media buffered with HEPES without NaHCO_3_ were different from the IC_50_ values of media buffered with NaHCO_3_. Therefore, the HEPES solution and absence of NaHCO_3_ seems to influence cell behavior, as we observed an increase of IC_50_ values in medium buffered at pH 7.4 compared with a normal medium at the same pH. To the best of our knowledge, there is no report about this issue.

Besides increased resistance to treatment, the low pHe in cancer is associated with many other malignant features. Some reports described the role of the acid tumor microenvironment in the metastization steps [57,58]. In addition, recent evidence showed the importance of the reverse pH gradient in tumor cell migration, controlled by pH regulators. The assembly of integrin filaments that drives membrane protrusion in migrating cells increases with a high intracellular pH and low extracellular pH [59]. It is also reported that V-ATPase and NHE1 colocalize with the actin cytoskeleton at the leading edge of migrating cells, indicating their involvement in cell migration [60,61,62]. Further, the optimal activity of MMPs and other proteolytic enzymes that degrade the extracellular matrix and support the dissemination of tumor cells occurs at low pHe [57,63]. We observed that lower pHe did not only increase the migratory capacity of MDA-MB-231 cells but also the invasion ability, probably due to the upregulation of MMP activity. Several MMPs have been shown to drive cell invasion through the basement membrane, such as MMP-2 and MMP-9, and their inhibition decreases the number of metastasis in in vivo models [64]. Our results showed that the acidic pHe increased MMP activity, namely MMP-9 in MDA-MB-231 cells.

The higher proliferation in acidic conditions can be due to a higher expression/activity of pH regulators. For this reason, we inhibited the activity of pH regulators using specific inhibitors in in vitro assays. Both cell lines were sensitive to all the compounds used and Conc. A cariporide CHC and AZ induced a reduction in cell viability to the same extent. Furthermore, inhibition of the pH regulators decreased the aggressive characteristics in the most aggressive cell line MDA-MB-231, namely by decreasing the migratory and invasive abilities. The acidic tumor microenvironment can also indirectly drive extracellular matrix proteolysis, as referred to above. Therefore, the stronger inhibition of these features in this cell line could be correlated with a reversion of abnormal pHe in cancer cells and the consequent lower activity of MMPs, namely MMP-2. In fact, we observed an increase in cancer cell pHe in the presence of these compounds, comparing to untreated cells, being this effect more evident in MDA-MB-231 cells (Additional file Table S4). In addition, it is described in the literature that inhibition of these pH regulators induce proton accumulation in the cell. This results in a decrease of pHi and increase of pHe, triggering an apoptotic cascade and a decrease in migration, invasion and drug resistance, thus reducing cancer aggressiveness (39).

pH regulator inhibitors can be used not only as primary cytotoxic agents but also to sensitize cancer cells to conventional chemotherapeutic agents. Our results demonstrated that all the compounds were able to improve doxorubicin’s toxic effects, with CHC and conc. A inducing a more evident effect (lower IC_50_ values for doxorubicin) in both cell lines (Figure 6). Similar results in the same field have already been reported, demonstrating the importance of pH manipulation in cancer therapy. Treatment with CHC led to sensitization to radiotherapy [36], induced tumor cell death and decreased tumor invasion in in vivo models [38]. In addition, Miranda-Gonçalves and coworkers demonstrated that MCT inhibition potentiates the cytotoxic effect of temozolomide in glioma cells [43]. Another report studied the influence of acidic vesicles in tumor cell conventional therapy resistance [65]. This resistance was disrupted by the V-ATPase inhibitor bafilomycin A, inducing an alkalinization of these compartments and drug activity by abolishing vesicle drug sequestration. Amith et al. also showed that NHE1 knockout sensitized MDA-MB-231 cells to paclitaxel [66]. Regarding conc. A or cariporide, to the best of our knowledge, their efficacy was not yet reported in combination with other drugs, as it was demonstrated here.

We also analyzed the expression of the pH regulators V-ATPase and CAXII in human breast cancer tissues due to their importance in cancer and to clarify the involvement of these pH regulators in breast cancer tissues. The results of the expression frequencies of MCTs were already published [20] and will be discussed here. We found overexpression of these two pH regulators in cancer tissues, namely at the cytoplasm for V-ATPase and PM for CAXII. The presence of V-ATPases in some types of cancer was associated with shorter overall survival and high-grade tumors, such as glioblastomas [67]. Concerning our results, no significant association was observed between V-ATPase and more aggressive features in breast cancer tissues, such as LN invasion. The role of CAXII in tumor aggressiveness is controversial. The expression of this protein is reported in human malignancies such as colorectal [68], cervical [69], renal [70] and other cancers [71,72]. However, despite its role in tumor reverse pH gradient, some studies associated CAXII expression with good prognostic factors in invasive breast cancer [73]. In contrast, other studies report its involvement with a poor prognosis in some types of cancers, such as oral cell carcinomas and glioma [74,75]. In this study, we observed in the immunohistochemistry assays an association with the most aggressive cancer subtype, triple-negative/basal subtype. Furthermore, almost 40% of cases present LN invasion. The lack of CAXII expression in MDA-MB-231 in in vitro assays is not in concordance with these results; however, this is most likely related to the singularity of this cell line.

Previous studies also demonstrated concomitant expression of some pH regulators, namely MCTs and CAIX, in the same series of TMAs used in this work [20], as well as in glioma tissues [37]. Another in vitro study showed simultaneous expression of V-ATPase and CAIX in breast cancer cell lines, but only in conditions of low oxygen levels [76]. Importantly, we evaluated for the first time the expression of these pH regulators, V-ATPase and CAXII, in human breast cancer tissues and investigated the association between them. We observed a close association between V-ATPase and CAXII expression, being these pH regulators co-expressed in the same clinical samples at the PM. Therefore, more than one pH regulator can be expressed at the same time, and a combined inhibition should be explored in future studies.

Concerning V-ATPase, we observed a cytoplasmic expression in clinical cases, but also PM expression in in vitro results. The clinical relevance of this aspect may represent an additional contribution to chemoresistance. The accumulation of acidic vesicles in the cytoplasm (e.g., lysosomes), acidified by proteins such as V-ATPase, may enhance drug sequestration and subsequent drug efflux through vesicular transport through the cell membrane and drug release to the extracellular space [77].

On the whole, our data support that different pH regulators expressed by cancer cells can be explored in the future as predictive biomarkers in breast cancer treatment. Further, disruption of proton dynamics in cancer cells can lead to the accumulation of intracellular protons to lethal levels and potentiate the effect of conventional therapeutic drugs, overcoming drug resistance.

## Figures and Tables

**Table 1:** Details about primary antibodies and conditions used in Western blot and immunofluorescence.

Protein	Dilution	Species	Reference, Supplier
NHE1	1:200	Rabbit	sc-28758, Santa Cruz Biotechnology
V-ATPase	1:200	Mouse	sc-374475, Santa Cruz Biotechnology
CAXII	1:200	Mouse	sc-374314, Santa Cruz Biotechnology
MCT1	1:200	Mouse	sc-365501, Santa Cruz Biotechnology
MCT4	1:500	Rabbit	sc-50329, Santa Cruz Biotechnology
α-tubulin	1:500	Rabbit	ab15246, Abcam

**Table 2:** Frequency of V-ATPase and CAXII expression in breast carcinomas tissues compared with normal breast epithelium.

	*n*	Positive (%)	*P*	PM (%)	*P*
**V-ATPase**			0.001 0.046		0.046
Normal breast epithelium	44	4 (9.1)		4 (9.1)	
Breast carcinoma	203	197 (97.0)		51 (25.9)	
**CAXII**					
Normal breast epithelium	40	0 (0.0)	0.032	0 (0.0)	0.001
Breast carcinoma	196	98 (50.0)		96 (98.0)	

**Table 3:** Association analysis of V-ATPase and CAXII with MCT1 and MCT4 expression in breast carcinoma samples.

	*n*	MCT1 Positive (%)	*P*	*n*	MCT4 Positive (%)	*P*
**V-ATPase**	123		0.876	120		0.444
Negative	87	18 (20.7)		84	4 (4.8)	
Positive	36	7 (19.4)		36	3 (8.3)	
**CAXII**	115		0.290	112		0.240
Negative	55	9 (16.4)		53	1 (1.9)	
Positive	60	15 (25.0)		59	4 (6.8)	

**Table 4:** Combination index (CI) values of PRIs and doxorubicin. PRIs were used at the respective IC_50_ concentration and combined with the IC_25_, IC_50_ and IC_75_ of doxorubicin.

	CI Values at					
	Dox IC_25_		Dox IC_50_		Dox IC_75_	
**Drug combination**	MDA	MCF-7	MDA	MCF-7	MDA	MCF-7
Conc. A + Dox	1.23 ± 0.034	1.66 ± 0.023	0.68 ± 0.012	0.74 ± 0.011	0.56 ± 0.010	0.62 ± 0.022
Cariporide + Dox	1.20 ± 0.022	1.34 ± 0.012	0.65 ± 0.003	0.84 ± 0.015	0.52 ± 0.007	0.76 ± 0.033
CHC + Dox	0.79 ± 0.045	0.94 ± 0.003	0.45 ± 0.005	0.77 ± 0.027	0.41 ± 0.009	0.64 ± 0.008
AZ + Dox	N.A±	0.71 ± 0.009	N.A	0.53 ± 0.007	N.A	0.51 ± 0.006

The effects on cell viability were determined using SRB cytotoxicity assay after 48 h, and the combination index (CI) values were calculated using CalcuSyn software (with CI values lower than 1 signifying synergism, values equal to 1 signifying additivity, values greater than 1 signifying antagonism). The CI values are presented as mean ± SD and were calculated from at least three independent experiments using a nonconstant ratio applying a fixed inhibitor concentration (value that corresponds to IC_50_ of each PRI). N.A: not applicable.

## Supplementary Materials

The following are available online at www.mdpi.com/xxx/s1..

## Abbreviations

AZ	Acetazolamide
CA	Carbonic anhydrase
Conc. A	Concanamycin A
CHC	α-cyano-4-hydroxycinnamate
ER	Estrogen receptor
GLUT1	glucose transporter 1
HER2	Human epidermal growth factor receptor 2
LN	lymph node
MCT	Monocarboxylate transporter
MMP	Metalloproteinase
NHE1	Sodium–hydrogen exchanger 1
PgR	Progesterone receptor
pHe	Extracellular pH
PM	Plasma membrane
PRIs	pH regulator inhibitors
TMAs	Tissue microarrays
TNBC	Triple negative breast cancer
V-ATPase	Vacuolar ATPase
