# Supplementary material for: Disruption of pH Dynamics Suppresses Proliferation and Potentiates Doxorubicin Cytotoxicity in Breast Cancer Cells
Source: Pharmaceutics. 2021 Feb 9;13(2):242. doi: 10.3390/pharmaceutics13020242 (PMC7916175; doi:10.3390/pharmaceutics13020242)
Supplement: Supplementary file 1 [file pharmaceutics-13-00242-s001.pdf]

*pharmaceutics*

# Supplementary Materials: Disruption of pH Dynamics Suppresses Proliferation and Potentiates Doxorubicin Cytotoxicity in Breast Cancer Cells

**Diana Tavares-Valente, Bárbara Sousa, Fernando Schmitt, Fátima Baltazar and Odília Queirós**

**Publisher's Note:** MDPI stays neutral with regard to jurisdictional claims in published maps and institutional affiliations.

**Copyright:** © 2021 by the authors. Submitted for possible open access publication under the terms and conditions of the Creative Commons Attribution (CC BY) license (http://creativecommons.org/licenses/by/4.0/).

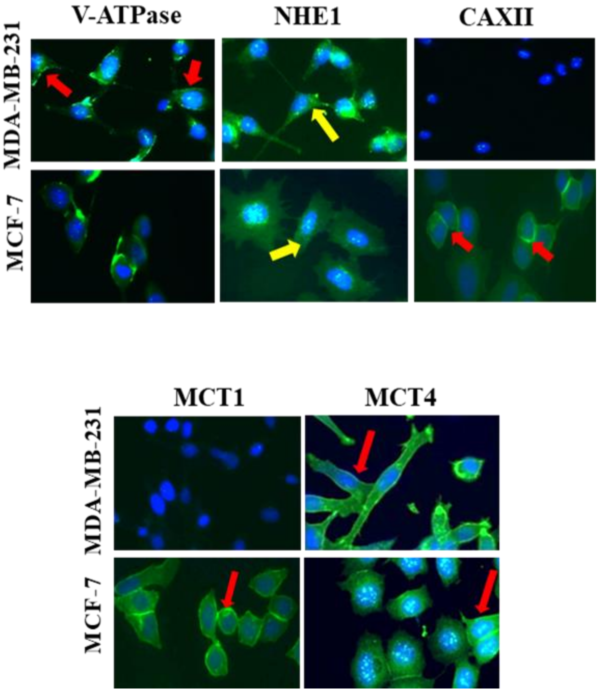


**Figure S1. Cellular localization of the different pH regulators determined by immunofluorescence.** The red arrows highlight PM expression and the yellow arrows cytoplasmic expression. Blue area corresponds to nuclei stained with DAPI. Images are in 100x magnification.

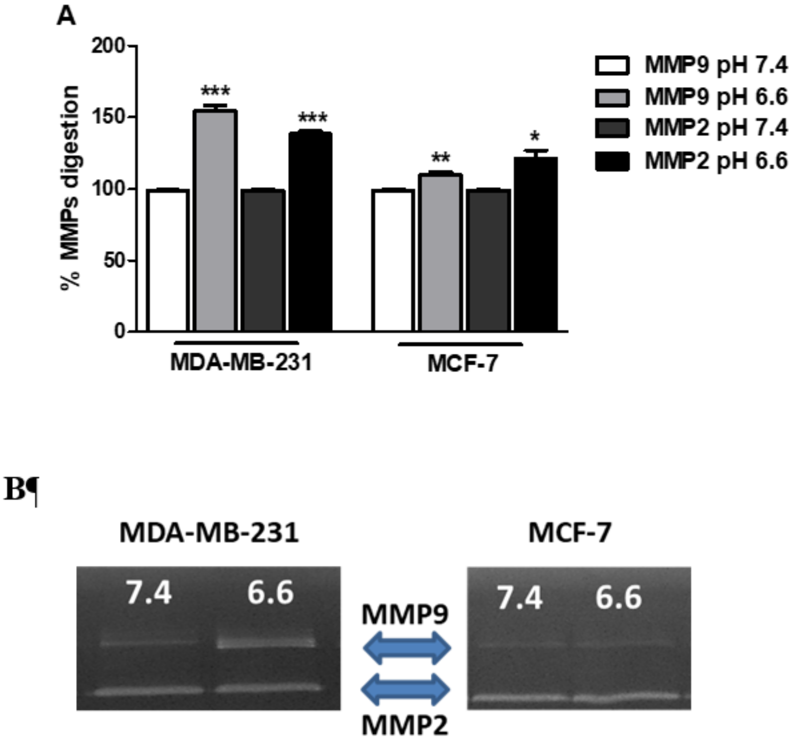


**Figure S2. Effect of extracellular pH on MMP activity assay in breast cancer cell lines. (A)** MMP9 and MMP2 activity in culture medium samples from breast cancer cells at different extracellular pH values, evaluated by zymography. **(B)** Representative pictures of gel digestion at different extracellular pH values. Results are expressed as mean ± SD of triplicates from three independent experiments. Significantly different between groups: **P* < 0.05; ***P* < 0.01 compared to control (pH 7.4). ns: non significant.

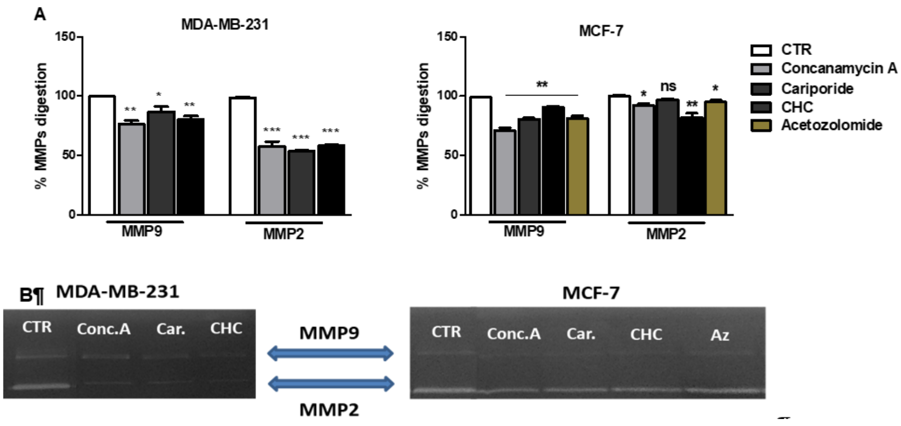


**Figure S3. Effect of pH regulator inhibition on MMP activity assay in breast cancer cell lines. (A)** MMP9 and MMP2 activity in culture medium samples from breast cancer cells in the presence of PRI, evaluated by zymography. **(B)** Representative pictures of gel digestion in the presence of PRI. Results are expressed as mean ± SD of triplicates from three independent experiments. Significantly different between groups: **P* < 0.05; ***P* < 0.01; ****P* < 0.001 compared to untreated cells (control).

**Table S1. Clinical, pathological and immunohistochemical characteristics of the 473 primary invasive breast carcinomas.** Characterization of the breast cancer series concerning age of the patients, tumor size, lymph-node metastasis, histological grade, molecular subtypes, ER, PgR, HER2 and Ki67 status, as well as expression of HIF-1α, GLUT1, CAIX, MCT1, MCT4 and CD147.

| | | | | |
|---|---|---|---|---|
| Clinico-pathological and molecular characteristics | **Age (years)** | Mean | 56.12 | |
| | | Min | 27 | |
| | | Max | 89 | |
| | | Missing | 2 | |
| | **Tumor size (mm)** | Mean | 31.6 | |
| | | Min | 5 | |
| | | Max | 150 | |
| | | Missing | 243 | |
| | | | **Frequency (n)** | **Percentage (%)** |
| | **Lymph-node metastasis** | Positive | 204 | 51 |
| | | Negative | 196 | 49 |
| | | Total | 400 | 100 |
| | | Missing | 73 | - |
| | **Histological grade** | I | 155 | 33.2 |
| | | II | 200 | 42.8 |
| | | III | 112 | 24 |
| | | Total | 467 | 100 |
| | | Missing | 6 | - |
| | **Molecular subtypes** | Luminal A | 262 | 57.3 |
| | | Luminal B | 14 | 3.1 |
| | | HER2 OE | 56 | 12.3 |
| | | Basal | 83 | 18.2 |
| | | Unclassified | 42 | 9.2 |
| | | Total | 457 | 100 |
| | | Missing | 16 | - |
| | **ER** | Positive | 275 | 58.4 |
| | | Negative | 196 | 41.6 |
| | | Total | 471 | 100 |
| | | Missing | 2 | - |
| | **PgR** | Positive | 177 | 37.9 |
| | | Negative | 290 | 62.1 |
| | | Total | 467 | 100 |
| | | Missing | 6 | |
| | **HER2** | Positive | 69 | 14.9 |
| | | Negative | 393 | 85.1 |
| | | Total | 462 | 100 |
| | | Missing | 11 | - |
| | **Ki67** | >20 | 98 | 39.5 |
| | | <20 | 150 | 60.5 |
| | | Total | 248 | 100 |
| | | Missing | 225 | - |
| Biomarkers | **HIF-1α** | Positive | 104 | 33 |
| | | Negative | 211 | 67.0 |
| | | Total | 315 | 100 |
| | | Missing | 158 | |
| | **GLUT1** | Positive | 140 | 42.8 |
| | | Negative | 187 | 57.2 |
| | | Total | 327 | 100 |
| | | Missing | 146 | - |

| | | | | |
|---|---|---|---|---|
| **Biomarkers** | **CAIX** | Positive | 66 | 20.9 |
| | | Negative | 250 | 79.1 |
| | | Total | 316 | 100 |
| | | Missing | 157 | - |
| | **MCT1** | Positive | 106 | 26 |
| | | Negative | 301 | 74 |
| | | Total | 407 | 100 |
| | | Missing | 66 | - |
| | **MCT4** | Positive | 69 | 16.5 |
| | | Negative | 350 | 83.5 |
| | | Total | 419 | 100 |
| | | Missing | 54 | - |
| | **CD147** | Positive | 24 | 11.1 |
| | | Negative | 193 | 88.9 |
| | | Total | 217 | 100 |
| | | Missing | 256 | - |

**Table S2.** Association of V-ATPase and CAXII with molecular characteristics and molecular subtypes in breast carcinoma samples.

| | | **V-ATPase** | | | ***P*** | **CAXII** | | | ***P*** |
|---|---|---|---|---|---|---|---|---|---|
| | | **Negative (%)** | **Positive (%)** | **Total** | | **Negative (%)** | **Positive (%)** | **Total** | |
| **Molecular Characteristics** | **ER** | | | | .001 | | | | .380 |
| | Negative | 47 (52.8) | 8 (22.2) | 55 (44.0) | | 20 (34.5) | 26 (43.3) | 46 (39.0) | |
| | Positive | 42 (47.2) | 28 (77.8) | 70 (56.0) | | 38 (65.5) | 34 (56.7) | 72 (61.0) | |
| | **Total** | 89 (71.2) | 36 (28.8) | 125 (100) | | 58 (49.0) | 60 (51.0) | 118 (100) | |
| | **PgR** | | | | .006 | | | | .010 |
| | Negative | 61 (70.9) | 16 (44.4) | 77 (63.1) | | 33 (58.9) | 37 (62.7) | 70 (60.9) | |
| | Positive | 25 (29.1) | 20 (55.6) | 45 (36.9) | | 23 (41.1) | 22 (37.3) | 45 (39.1) | |
| | **Total** | 86 (70.5) | 36 (29.5) | 122 (100) | | 56 (48.7) | 59 (51.3) | 115 (100) | |
| | **HER2** | | | | .003 | | | | .380 |
| | Negative | 62 (71.3) | 33 (94.3) | 95 (77.9) | | 38 (65.5) | 48 (82.8) | 91 (60.9) | |
| | Positive | 25 (2.7) | 2 (5.7) | 27 (22.1) | | 20 (41.1) | 10 (17.2) | 25 (21.6) | |
| | **Total** | 87 (71.3) | 35 (28.7) | 122 (100) | | 58 (50.0) | 58 (50.0) | 116 (100) | |
| | **Ki67** | | | | .080 | | | | .040 |
| | <10 | 52 (59.8) | 16 (45.7) | 68 (55.7) | | 28 (48.3) | 37 (64.9) | 65 (56.5) | |
| | 10-20 | 8 (9.2) | 1 (2.9) | 9 (7.4) | | 8 (13.8) | 3 (5.3) | 11 (9.6) | |
| | >20 | 27 (31.0) | 18 (51.4) | 45 (36.9) | | 22 (37.9) | 17 (29.8) | 39 (33.9) | |
| | **Total** | 87 (71.3) | 35 (28.7) | 122 (100) | | 58 (50.4) | 57 (49.6) | 115 (100) | |
| | **LN invasion** | | | | .656 | | | | .010 |
| | No | 38 (46.9) | 13 (52.0) | 51 (48.1) | | 17 (34.7) | 29 (61.7) | 46 (47.9) | |
| | Yes | 43 (53.1) | 12 (48.0) | 55 (51.9) | | 32 (65.3) | 18 (38.3) | 50 (52.1) | |
| | **Total** | 81 (76.4) | 25 (23.6) | 106 (100) | | 49 (51.0) | 47 (49.0) | 96 (100) | |
| **Molecular** | **Luminal A** | 37 (42.5) | 27 (77.1) | 64 (52.5) | .006 | 31 (54.4) | 32 (55.2) | 63 (54.8) | .010 |
| | **Luminal B** | 4 (4.6) | 0 (0.0) | 4 (3.3) | | 6 (10.5) | 1 (1.7) | 7 (6.1) | |
| | **HER2 overexpressing** | 21 (24.1) | 2 (5.7) | 23 (18.9) | | 9 (15.8) | 9 (15.5) | 18 (15.7) | |
| | **Basal** | 14 (16.1) | 5 (14.3) | 19 (15.6) | | 6 (10.5) | 11 (19.0) | 17 (14.8) | |
| | **Unclassified** | 11 (12.6) | 1 (2.9) | 12 (9.8) | | 5 (8.8) | 5 (8.6) | 10 (8.7) | |
| | **Total** | 87 (71.3) | 35 (28.7) | 122 (100) | | 57 (49.6) | 58 (50.4) | 115 (100) | |

*It was considered only the plasma membrane expression of both pH regulators.

**Table S3.** Association of V-ATPase and CAXII with biomarkers in breast carcinoma samples.

| Biomarkers | V-ATPase Negative (%) | V-ATPase Positive (%) | V-ATPase *Total* | *P* | CAXII Negative (%) | CAXII Positive (%) | CAXII *Total* | *P* |
|---|---|---|---|---|---|---|---|---|
| **HIF1** | | | | **.687** | | | | **.362** |
| Negative | 23 (36.5) | 9 (32.1) | 32 (35.2) | | 17 (37.8) | 10 (28.6) | 27 (33.8) | |
| Positive | 40 (63.5) | 19 (67.9) | 59 (64.8) | | 28 (62.2) | 25 (71.4) | 53 (66.3) | |
| **Total** | 63 (69.2) | 28 (30.8) | 91 (100) | | 45 (56.3) | 35 (43.7) | 80 (100) | |
| **GLUT1** | | | | **.070** | | | | **.264** |
| Negative | 16 (61.5) | 4 (30.8) | 20 (51.3) | | 12 (66.7) | 4 (30.8) | 16 (51.6) | |
| Positive | 10 (38.5) | 9 (69.2) | 19 (48.7) | | 6 (33.3) | 9 (69.2) | 15 (48.4) | |
| **Total** | 26 (66.7) | 13 (33.3) | 39 (100) | | 18 (58.0) | 13 (42.0) | 31 (100) | |
| **CAIX** | | | | **.363** | | | | **.213** |
| Negative | 19 (73.1) | 7 (58.3) | 26 (68.4) | | 15 (78.9) | 7 (58.3) | 22 (71.0) | |
| Positive | 7 (26.9) | 5 (41.7) | 12 (31.6) | | 4 (21.1) | 5 (41.7) | 9 (29.0) | |
| **Total** | 26 (68.4) | 12 (31.6) | 38 (100) | | 19 (61.3) | 12 (38.7) | 31 (100) | |
| **CD147** | | | | **.342** | | | | **.301** |
| Negative | 78 (91.8) | 31 (86.1) | 109 (90.1) | | 52 (96.3) | 53 (91.4) | 105 (93.8) | |
| Positive | 7 (8.2) | 5 (13.9) | 12 (9.9) | | 2 (3.7) | 5 (8.6) | 7 (6.3) | |
| **Total** | 85 (70.2) | 36 (29.8) | 121 (100) | | 54 (48.2) | 58 (51.8) | 112 (100) | |
| **V-ATPase** | | | | - | | | | **.030** |
| Negative | - | - | - | | 41 (83.7) | 30 (63.8) | 71 (74.0) | |
| Positive | - | - | - | | 8 (16.3) | 17 (36.2) | 25 (26.0) | |
| Total | - | - | - | | 49 (51.0) | 47 (49.0) | 96 (100) | |

*It was considered only the plasma membrane expression of both pH regulators.

**Table S4. pHe values of culture media measured after 48 hours after PRI $IC_{50}$ treatment.** The ΔpH corresponds to the difference between the pHe after treatment and the pHe without treatment, after the respective time of incubation. Results represent the mean + SD of at least three independent experiments.

| | W/o compound | Conc.A | Cariporide | AZ | CHC | ΔpH |
|---|---|---|---|---|---|---|
| **MDA-MB-231** | 5.4 ± 0.7 | 6.2 ± 0.2 | 6.5 ± 0.2 | - | 6.4 ± 0.6 | Conc. A: 0.9<br>Cariporide: 1.1<br>CHC: 1.0 |
| **MCF-7** | 5.8 ± 0.2 | 6.2 ± 0.9 | 6.4 ± 0.2 | 6.6 ± 0.8 | 6.0 ± 0.2 | Conc. A: 0.4<br>Cariporide: 0.6<br>AZ:0.8<br>CHC: 0.2 |
